# Supplementary material for: Prevalence of COVID-19 in adolescents and youth compared with older adults in states experiencing surges
Source: PLoS One. 2021 Mar 10;16(3):e0242587. doi: 10.1371/journal.pone.0242587 (PMC7946189; doi:10.1371/journal.pone.0242587)
Supplement: S1 Appendix — (ZIP) [file pone.0242587.s001.zip › S1_Appendix/page 1.pdf]

Appendix S1, Supplemental material for the manuscript:

"Prevalence of COVID-19 in Adolescents and Youth Compared with Older Adults in States Experiencing Surges"

by B Rumain, M Schneiderman, A Geliebter

Methodological Details

This supplementary material is provided by the authors to give readers additional source information about the data used in the study.

S1.1. Case Data by State Websites:

(i) South Dakota: https://doh.sd.gov/news/coronavirus.aspx [Retrieved Sept. 4, 2020]

(ii) Tennessee: https://www.tn.gov/content/tn/health/cedep/ncov/data.html [Retrieved August 12, 2020]

(iii) Utah: https://coronavirus.utah.gov/case-counts/[Retrieved August 18, 2020]

(iv) Florida: https://floridahealthcovid19.gov/ [retrieved July 19, 2020]

(v) Kansas: https:// www.coronavirus.kdheks.gov/160/COVID-19-in-Kansas [Retrieved Aug 23, 2020]

(vi)Missouri: Cases by Age | COVID-19 Outbreak | Health & Senior Services (mo.gov) [Retrieved August 7, 2020]

S1.2. Demographic Data by Age and by State Websites:

(i) South Dakota: https://www.sdstate.edu/sociology-rural-studies/census-data-center/population-change

(ii) Tennessee: https://www.tn.gov/content/dam/tn/health/documents/population/TN-Population-by-AgeGrp-Sex-Race-Ethnicity-2019.pdf

(iii) Utah: https://gardner.utah.edu/wp-content/uploads/State-of-Utah-Demographic-Profile-2010-2018.pdf
