# Supplementary material for: Prevalence of COVID-19 in adolescents and youth compared with older adults in states experiencing surges
Source: PLoS One. 2021 Mar 10;16(3):e0242587. doi: 10.1371/journal.pone.0242587 (PMC7946189; doi:10.1371/journal.pone.0242587)
Supplement: S1 Appendix — (ZIP) [file pone.0242587.s001.zip › S1_Appendix/page 10.pdf]

## Lorem Ipsum

Lorem ipsum dolor sit amet, consectetur adipiscing elit. Mauris maximus fringilla ligula, in malesuada erat tempor ac. Quisque dapibus posuere turpis, vel aliquam massa vehicula non.

**Table G. South Dakota Demographics by Age**

| Age | 2020 | 2025 |
|---|---|---|
| 0-4 | 60089 | 60941 |
| 5-9 | 58068 | 60533 |
| 10-14 | 60651 | 58650 |
| 15-19 | 56625 | 61169 |
| 20-24 | 54948 | 56947 |
| 25-29 | 58423 | 55332 |
| 30-34 | 58336 | 58765 |
| 35-39 | 56111 | 58593 |
| 40-44 | 50260 | 56250 |
| 45-49 | 46109 | 50294 |
| 50-54 | 47491 | 45904 |
| 55-59 | 56783 | 47102 |
| 60-64 | 57344 | 55432 |
| 65-69 | 50577 | 54920 |
| 70-74 | 38624 | 46897 |
| 75-79 | 26368 | 34250 |
| 80-84 | 18985 | 21837 |
| 85+ | 33655 | 38932 |
| Total | 889447 | 922748 |

From South Dakota State University, Open PRAIRIE (Public Research Access Institutional Repository and Informational Exchange)

https://www.sdstate.edu/sociology-rural-studies/census-data-center/population-change



**test-test-1** This is a preview of your figure rendered on a simulated PLOS journal page.

Maecenas ac est sit amet odio sollicitudin euismod. In risus odio, convallis a neque ac, varius ultricies arcu. Vestibulum et quam iaculis, ultricies odio et, molestie magna. Suspendisse vehicula purus id turpis eleifend, et convallis dui dignissim. Praesent tempus elit a metus sollicitudin, sed fringilla nulla porttitor. Nullam in tempus massa. Nunc maximus magna massa, nec volutpat risus rhoncus ut. Fusce quis ante sem. Aenean nulla nibh, tempus sit amet rhoncus at, eleifend vel risus. Sed dictum, sem ultrices elementum pharetra, lacus diam volutpat orci, scelerisque semper dui lacus ut enim.

Suspendisse in nunc id lacus commodo consequat. Proin semper aliquam varius. Fusce vitae neque aliquam nisi ultrices sodales vitae ut enim. Vivamus nec dictum ipsum. Sed condimentum ante eu urna tincidunt tincidunt. In ac lacus nec ipsum viverra volutpat posuere vel lacus. Class aptent taciti sociosqu ad litora torquent per conubia nostra, per inceptos himenaeos. Morbi rhoncus ipsum quis lorem hendrerit, at vulputate massa tempus. Ut arcu nisl, gravida vitae risus ultricies, porta venenatis massa. Cras dignissim, enim at faucibus aliquam, sapien nisl eleifend dolor, vel mollis nulla nisi id ipsum. Pellentesque vehicula ultricies risus sit amet faucibus. Praesent sit amet mi ac est faucibus accumsan. Praesent pulvinar sit amet orci auctor feugiat.
