# Supplementary material for: Prevalence of COVID-19 in adolescents and youth compared with older adults in states experiencing surges
Source: PLoS One. 2021 Mar 10;16(3):e0242587. doi: 10.1371/journal.pone.0242587 (PMC7946189; doi:10.1371/journal.pone.0242587)
Supplement: S1 Appendix — (ZIP) [file pone.0242587.s001.zip › S1_Appendix/page 11.pdf]

Lorem Ipsum

Lorem ipsum dolor sit amet, consectetur adipiscing elit. Mauris maximus fringilla ligula, in malesuada erat tempor ac. Quisque dapibus posuere turpis, vel aliquam massa vehicula non.

Table H. Tennessee Population Counts by Age Group, Sex, Race and Ethnicity, Estimates 2019

TENNESSEE

Age Group	Total Pop	Total Male	Total Female	Total White	White Male	White Female	Total Black	Black Male	Black Female	Total Other	Other Male	Other Female	Total Hispanic	Hispanic Male	Hispanic Female
All Ages	6,829,116	3,332,198	3,496,918	5,354,401	2,630,482	2,723,919	1,164,407	550,189	614,218	310,308	151,527	158,781	391,434	205,513	185,921
Infants	82,167	41,910	40,257	59,996	30,859	29,137	15,901	7,912	7,989	6,270	3,139	3,131	9,182	4,620	4,562
1 to 4	326,444	166,903	159,541	236,139	121,401	114,738	65,162	32,789	32,373	25,143	12,713	12,430	36,098	18,411	17,687
5 to 9	413,380	211,440	201,940	299,164	153,486	145,678	83,316	42,292	41,024	30,900	15,662	15,238	44,048	22,674	21,374
10 to 14	431,289	219,753	211,536	318,077	162,645	155,432	83,659	42,355	41,304	29,553	14,753	14,800	43,180	22,046	21,134
15 to 19	424,285	216,475	207,810	315,407	162,146	153,261	83,149	41,651	41,498	25,729	12,678	13,051	34,402	17,830	16,572
20 to 24	444,932	225,079	219,853	330,252	167,808	162,444	90,346	45,123	45,223	24,334	12,148	12,186	29,814	15,359	14,455
25 to 29	497,285	249,334	247,951	370,325	186,695	183,630	102,229	50,355	51,874	24,731	12,284	12,447	30,479	16,406	14,073
30 to 34	452,368	224,231	228,137	344,579	172,149	172,430	85,247	40,923	44,324	22,542	11,159	11,383	29,515	16,070	13,445
35 to 39	435,526	214,783	220,743	337,091	168,365	168,726	77,208	35,929	41,279	21,227	10,489	10,738	31,017	16,965	14,052
40 to 44	412,110	202,742	209,368	322,895	161,198	161,697	70,317	32,547	37,770	18,898	8,997	9,901	27,478	15,212	12,266
45 to 49	437,984	214,893	223,091	349,282	173,922	175,360	71,569	32,941	38,628	17,133	8,030	9,103	22,223	11,959	10,264
50 to 54	433,274	212,008	221,266	349,981	173,487	176,494	68,976	31,696	37,280	14,317	6,825	7,492	16,691	9,071	7,620
55 to 59	460,265	220,966	239,299	375,352	181,912	193,440	71,772	32,782	38,990	13,141	6,272	6,869	12,666	6,640	6,026
60 to 64	434,395	205,268	229,127	357,184	170,939	186,245	65,672	29,037	36,635	11,539	5,292	6,247	8,950	4,653	4,297
65 to 69	373,777	174,032	199,745	313,456	147,932	165,524	51,296	22,064	29,232	9,025	4,036	4,989	5,918	2,982	2,936
70 to 74	307,621	142,343	165,278	267,097	125,009	142,088	33,691	14,137	19,554	6,833	3,197	3,636	4,131	2,029	2,102
75 to 79	209,205	93,712	115,493	184,758	83,889	100,869	20,256	7,850	12,406	4,191	1,973	2,218	2,473	1,184	1,289
80 to 84	131,040	54,453	76,587	115,741	48,875	66,866	12,697	4,474	8,223	2,602	1,104	1,498	1,498	700	798
85 plus	121,769	41,873	79,896	107,625	37,765	69,860	11,944	3,332	8,612	2,200	776	1,424	1,671	702	969

Note: People who are Hispanic may be of any race.

Source: Tennessee Department of Health, Division of PHA; based on interpolated data from the U.S. Census, Annual Estimates of the Resident Population: April 1, 2010 to July 1, 2019.

Appendix S1_202102222145-11.tif This is a preview of your figure rendered on a simulated PLOS journal page.

Maecenas ac est sit amet odio sollicitudin euismod. In risus odio, convallis a neque ac, varius ultricies arcu. Vestibulum et quam iaculis, ultricies odio et, molestie magna. Suspendisse vehicula purus id turpis eleifend, et convallis dui dignissim. Praesent tempus elit a metus sollicitudin, sed fringilla nulla porttitor. Nullam in tempus massa. Nunc maximus magna massa, nec volutpat risus rhoncus ut. Fusce quis ante sem. Aenean nulla nibh, tempus sit amet rhoncus at, eleifend vel risus. Sed dictum, sem ultrices elementum pharetra, lacus diam volutpat orci, scelerisque semper dui lacus ut enim.

Suspendisse in nunc id lacus commodo consequat. Proin semper aliquam varius. Fusce vitae neque aliquam nisi ultrices sodales vitae ut enim. Vivamus nec dictum ipsum. Sed condimentum ante eu urna tincidunt tincidunt. In ac lacus nec ipsum viverra volutpat posuere vel lacus. Class aptent taciti sociosqu ad litora torquent per conubia nostra, per inceptos himenaeos. Morbi rhoncus ipsum quis lorem hendrerit, at vulputate massa tempus. Ut arcu nisl, gravida vitae risus ultricies, porta venenatis massa. Cras dignissim, enim at faucibus aliquam, sapien nisl eleifend dolor, vel mollis nulla nisi id ipsum. Pellentesque vehicula ultricies risus sit amet faucibus. Praesent sit amet mi ac est faucibus accumsan. Praesent pulvinar sit amet orci auctor feugiat.

Phasellus vitae congue est. Duis rutrum iaculis nunc, sed sollicitudin neque eleifend nec. Pellentesque ac nisi eget tortor imperdiet sagittis ut in orci. Mauris porta convallis euismod. Donec in ultricies urna, nec interdum lectus. Nullam sit amet finibus augue, eget rutrum metus. Nam faucibus, urna ac finibus eleifend, neque nisi lobortis ante, at pharetra purus purus sed urna. Curabitur sit amet dui at enim porta posuere non vehicula ligula. Suspendisse potenti. Vestibulum arcu magna, vulputate a massa ac, molestie tincidunt dui.

Donec id tempus lacus, sed tristique nulla. Nullam rutrum risus ut pharetra porttitor. Nam mattis dolor erat, sed volutpat est mattis sed. Suspendisse eu porta tellus. Cras gravida velit sed maximus fermentum. Fusce vitae metus commodo, sagittis nunc sed, faucibus nunc. Integer iaculis quam mattis, luctus neque in, viverra magna. Nulla rhoncus feugiat orci, quis posuere ligula ornare at. Integer vel sagittis risus. Donec semper metus nec finibus accumsan. Mauris sit amet suscipit ante. Aliquam accumsan, nisl vitae vulputate elementum, turpis nibh varius urna, vel bibendum nulla nunc ac quam. Aenean malesuada egestas maximus. Pellentesque faucibus, odio at tincidunt ullamcorper, eros nisi pellentesque mi, non blandit sapien neque quis lectus.
