# Supplementary material for: Prevalence of COVID-19 in adolescents and youth compared with older adults in states experiencing surges
Source: PLoS One. 2021 Mar 10;16(3):e0242587. doi: 10.1371/journal.pone.0242587 (PMC7946189; doi:10.1371/journal.pone.0242587)
Supplement: S1 Appendix — (ZIP) [file pone.0242587.s001.zip › S1_Appendix/page 12.pdf]

## Lorem Ipsum

Lorem ipsum dolor sit amet, consectetur adipiscing elit. Mauris maximus fringilla ligula, in malesuada erat tempor ac. Quisque dapibus posuere turpis, vel aliquam massa vehicula non.

Table I. Utah Demographics by Age, Sex, Race, and Ethnicity

*Demographic Profile*

November 2019

Kem C. Gardner
POLICY INSTITUTE
THE UNIVERSITY OF UTAH
DAVID ECCLES SCHOOL OF BUSINESS

# State of Utah Population by Age, Sex, Race and Ethnicity, 2010–2018

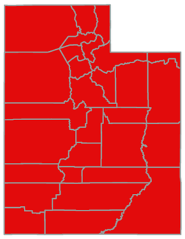

Utah's 2018 population was estimated at 3,116,647. Between 2010 and 2018, it grew by 394,274, representing an average annual percentage growth rate of 1.7 percent. The state remains relatively young with 29.7 percent of the population being preschool or school age (under 18), compared to 10.9 percent of retirement age (65 and older). However, like the rest of the nation, the state continues to age rapidly as birth rates drop, people live longer, and Baby Boomers reach retirement ages. Between 2010 and 2018, median age rose from to 29.3 to 31.4. The retirement age population grew faster than any other age group, averaging 4.1 percent growth annually. For all ages, the state is expected to continue growing, in large part due to above-average birth rates and positive net migration. Utah has emerged as a consistent net in-migration state because of an expanding economy, wide array of outdoor opportunities, and vast network of global cultural and economic interconnections.

### How do these Estimates Differ from Census Bureau Estimates?

The Kem C. Gardner Policy Institute produces an annual suite of detailed population estimates. These control to official numbers published by the Utah Population Committee. The Census Bureau produces similar population estimates that are used for federal

### Utah State Population Pyramid, 2018

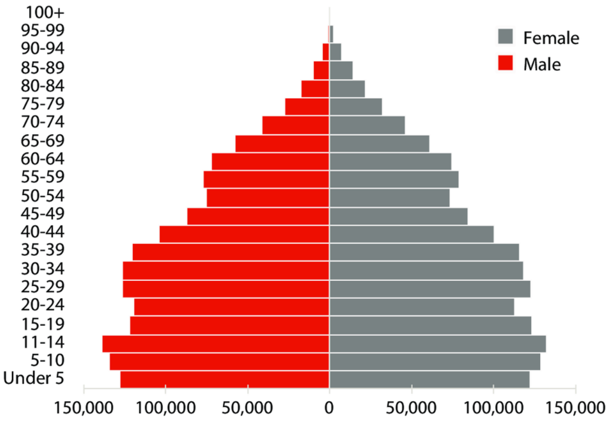


Source: Kem C Gardner Policy Institute

### Utah State Population, 2018

| Age | Male | Female | Total | Share | Sex Ratio |
|---|---|---|---|---|---|
| **Total** | **1,593,103** | **1,573,543** | **3,166,647** | **100.0%** | **1.01** |
| Under 5 | 128,101 | 122,214 | 250,315 | 7.9% | 1.05 |
| 5-10 | 134,517 | 128,545 | 263,061 | 8.3% | 1.05 |
| 11-14 | 139,110 | 132,046 | 271,156 | 8.6% | 1.05 |
| 15-19 | 122,118 | 123,286 | 245,404 | 7.7% | 0.99 |
| 20-24 | 119,605 | 113,066 | 232,671 | 7.3% | 1.06 |
| 25-29 | 126,593 | 122,694 | 249,287 | 7.9% | 1.03 |
| 30-34 | 126,637 | 118,094 | 244,731 | 7.7% | 1.07 |
| 35-39 | 120,695 | 115,857 | 236,552 | 7.5% | 1.04 |
| 40-44 | 103,905 | 100,272 | 204,177 | 6.4% | 1.04 |
| 45-49 | 86,978 | 84,352 | 171,330 | 5.4% | 1.03 |
| 50-54 | 75,364 | 73,742 | 149,107 | 4.7% | 1.02 |
| 55-59 | 77,289 | 78,828 | 156,117 | 4.9% | 0.98 |
| 60-64 | 72,050 | 74,407 | 146,457 | 4.6% | 0.97 |
| 65-69 | 57,617 | 61,273 | 118,890 | 3.8% | 0.94 |
| 70-74 | 41,660 | 46,298 | 87,958 | 2.8% | 0.90 |
| 75-79 | 27,396 | 32,314 | 59,710 | 1.9% | 0.85 |
| 80-84 | 17,370 | 21,950 | 39,320 | 1.2% | 0.79 |
| 85-89 | 10,171 | 14,121 | 24,292 | 0.8% | 0.72 |
| 90-94 | 4,582 | 7,407 | 11,990 | 0.4% | 0.62 |
| 95-99 | 1,201 | 2,406 | 3,607 | 0.1% | 0.50 |
| 100+ | 144 | 370 | 514 | 0.0% | 0.39 |
| School Age (5-17) | 353,583 | 337,148 | 690,731 | 21.8% | 1.05 |
| College Age (18-24) | 161,766 | 159,795 | 321,561 | 10.2% | 1.01 |
| Working Age (18-64) | 951,277 | 928,042 | 1,879,319 | 59.3% | 1.03 |
| Under 18 | 481,684 | 459,362 | 941,046 | 29.7% | 1.05 |
| 50+ | 384,846 | 413,117 | 797,963 | 25.2% | 0.93 |
| Retirement Age (65+) | 160,142 | 186,140 | 346,282 | 10.9% | 0.86 |
| 85+ | 16,099 | 24,304 | 40,403 | 1.3% | 0.66 |
| Median Age | 31.1 | 31.9 | 31.4 | - | - |

| | Ratio |
|---|---|
| Youth Dependency Ratio | 50.1 |
| Retirement Dependency Ratio | 18.4 |
| Total Dependency Ratio | 68.5 |

Source: Kem C Gardner Policy Institute

**test-test-1** This is a preview of your figure rendered on a simulated PLOS journal page.

Maecenas ac est sit amet odio sollicitudin euismod. In risus odio, convallis a neque ac, varius ultricies arcu. Vestibulum et quam iaculis, ultricies odio et, molestie magna. Suspendisse vehicula purus id turpis eleifend, et convallis dui dignissim. Praesent tempus elit a metus sollicitudin, sed fringilla nulla porttitor. Nullam in tempus massa. Nunc maximus magna massa, nec volutpat risus rhoncus ut. Fusce quis ante sem. Aenean nulla nibh, tempus sit amet rhoncus at, eleifend vel risus. Sed dictum, sem ultrices elementum pharetra, lacus diam volutpat orci, scelerisque semper dui lacus ut enim.

Suspendisse in nunc id lacus commodo consequat. Proin semper aliquam varius. Fusce vitae neque aliquam nisi ultrices sodales vitae ut enim. Vivamus nec dictum ipsum. Sed condimentum ante eu urna tincidunt tincidunt. In ac lacus nec ipsum viverra volutpat posuere vel lacus. Class aptent taciti sociosqu ad litora torquent per conubia nostra, per inceptos himenaeos. Morbi rhoncus ipsum quis lorem hendrerit, at vulputate massa tempus. Ut arcu nisl, gravida vitae risus ultricies, porta venenatis massa. Cras dignissim, enim at faucibus aliquam, sapien nisl eleifend dolor, vel mollis nulla nisi id ipsum. Pellentesque vehicula ultricies risus sit amet faucibus. Praesent sit amet mi ac est faucibus accumsan. Praesent pulvinar sit amet orci auctor feugiat.
