# Supplementary material for: Prevalence of COVID-19 in adolescents and youth compared with older adults in states experiencing surges
Source: PLoS One. 2021 Mar 10;16(3):e0242587. doi: 10.1371/journal.pone.0242587 (PMC7946189; doi:10.1371/journal.pone.0242587)
Supplement: S1 Appendix — (ZIP) [file pone.0242587.s001.zip › S1_Appendix/page 13.pdf]

Lorem Ipsum

Lorem ipsum dolor sit amet, consectetur adipiscing elit. Mauris maximus fringilla ligula, in malesuada erat tempor ac. Quisque dapibus posuere turpis, vel aliquam massa vehicula non.

Table J. **FLORIDA POPULATION BY AGE GROUP**

Age Group	1960 Census	1970 Census	1980 Census	1990 Census	2000 Census	2010 Census	2020 Projected	2030 Projected	2040 Projected
**0-4**	**542,221**	**501,172**	**570,247**	**873,033**	**945,853**	**1,073,506**	**1,163,815**	**1,287,190**	**1,370,468**
5-9	490,152	605,715	621,565	809,316	1,031,748	1,080,255	1,145,547	1,293,604	1,401,882
10-14	434,842	643,011	685,048	746,353	1,057,055	1,130,847	1,230,501	1,308,363	1,431,964
15-17	214,279	359,229	482,909	455,165	611,794	717,483	744,435	775,569	865,263
**5-17**	**1,139,273**	**1,607,955**	**1,789,522**	**2,010,834**	**2,700,597**	**2,928,585**	**3,120,483**	**3,377,536**	**3,699,109**
18-19	123,403	217,541	328,473	348,626	402,302	510,899	524,005	545,831	607,084
20-24	296,631	496,521	811,469	878,855	928,334	1,228,758	1,286,875	1,422,739	1,491,787
**18-24**	**420,034**	**714,062**	**1,139,942**	**1,227,481**	**1,330,636**	**1,739,657**	**1,810,880**	**1,968,570**	**2,098,871**
25-29	296,161	403,103	739,949	1,055,088	995,394	1,179,227	1,363,731	1,433,092	1,479,248
30-34	326,983	358,666	671,630	1,062,270	1,088,782	1,110,318	1,349,179	1,444,369	1,581,318
35-39	338,734	355,416	554,259	951,471	1,261,083	1,178,467	1,299,067	1,534,665	1,600,911
40-44	313,468	391,458	484,550	851,931	1,224,256	1,252,787	1,203,452	1,477,201	1,579,602
**25-44**	**1,275,346**	**1,508,643**	**2,450,388**	**3,920,760**	**4,569,515**	**4,720,799**	**5,215,429**	**5,889,327**	**6,241,079**
45-49	293,431	394,448	471,308	690,766	1,085,434	1,401,202	1,244,141	1,387,364	1,634,158
50-54	262,798	367,550	517,915	591,856	984,102	1,340,291	1,302,262	1,268,910	1,546,135
55-59	240,928	347,341	555,238	586,879	821,531	1,202,418	1,470,728	1,325,864	1,470,275
60-64	222,411	358,917	564,696	680,524	737,506	1,135,250	1,443,760	1,428,312	1,378,675
**45-64**	**1,019,568**	**1,468,256**	**2,109,157**	**2,550,025**	**3,628,573**	**5,079,161**	**5,460,891**	**5,410,450**	**6,029,243**
65-69	224,162	357,541	579,058	737,123	727,505	959,233	1,291,871	1,617,507	1,445,548
70-74	165,552	289,140	480,167	627,705	724,697	768,707	1,149,780	1,450,873	1,433,597
75-79	96,677	188,217	329,229	483,538	616,703	615,514	845,997	1,155,594	1,435,018
80-84	44,452	96,896	181,878	302,103	407,444	482,023	578,645	879,520	1,109,229
85 and up	24,275	57,565	117,373	205,469	331,301	434,125	598,876	835,999	1,219,230
**65 and up**	**555,118**	**989,359**	**1,687,705**	**2,355,938**	**2,807,650**	**3,259,602**	**4,465,169**	**5,939,493**	**6,642,622**
**TOTAL**	**4,951,560**	**6,789,447**	**9,746,961**	**12,938,071**	**15,982,824**	**18,801,310**	**21,236,667**	**23,872,566**	**26,081,392**

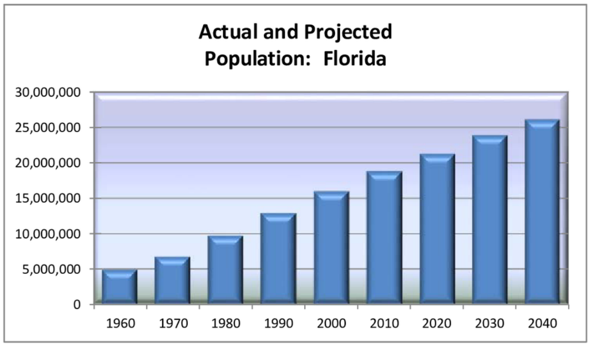

Source: Florida Demographic Estimating Conference, February 2015 and the University of Florida, Bureau of Economic and Business Research, Florida Population Studies, Bulletin 172, June 2015

Appendix S1_202102222145-13.tif This is a preview of your figure rendered on a simulated PLOS journal page.

Maecenas ac est sit amet odio sollicitudin euismod. In risus odio, convallis a neque ac, varius ultricies arcu. Vestibulum et quam iaculis, ultricies odio et, molestie magna. Suspendisse vehicula purus id turpis eleifend, et convallis dui dignissim. Praesent tempus elit a metus sollicitudin, sed fringilla nulla porttitor. Nullam in tempus massa. Nunc maximus magna massa, nec volutpat risus rhoncus ut. Fusce quis ante sem. Aenean nulla nibh, tempus sit amet rhoncus at, eleifend vel risus. Sed dictum, sem ultrices elementum pharetra, lacus diam volutpat orci, scelerisque semper dui lacus ut enim.

Suspendisse in nunc id lacus commodo consequat. Proin semper aliquam varius. Fusce vitae neque aliquam nisi ultrices sodales vitae ut enim. Vivamus nec dictum ipsum. Sed condimentum ante eu urna tincidunt tincidunt. In ac lacus nec ipsum viverra volutpat posuere vel lacus. Class aptent taciti sociosqu ad litora torquent per conubia nostra, per inceptos himenaeos. Morbi rhoncus ipsum quis lorem hendrerit, at vulputate massa tempus. Ut arcu nisl, gravida vitae risus ultricies, porta venenatis massa. Cras dignissim, enim at faucibus aliquam, sapien nisl eleifend dolor, vel mollis nulla nisi id ipsum. Pellentesque vehicula ultricies risus sit amet faucibus. Praesent sit amet mi ac est faucibus accumsan. Praesent pulvinar sit amet orci auctor feugiat.

Phasellus vitae congue est. Duis rutrum iaculis nunc, sed sollicitudin neque eleifend nec. Pellentesque ac nisi eget tortor imperdiet sagittis ut in orci. Mauris porta convallis euismod. Donec in ultricies urna, nec interdum lectus. Nullam sit amet finibus augue, eget rutrum metus. Nam faucibus, urna ac finibus eleifend, neque nisi lobortis ante, at pharetra purus purus sed urna. Curabitur sit amet dui at enim porta posuere non vehicula ligula. Suspendisse potenti. Vestibulum arcu magna, vulputate a massa ac, molestie tincidunt dui.

Donec id tempus lacus, sed tristique nulla. Nullam rutrum risus ut pharetra porttitor. Nam mattis dolor erat, sed volutpat est mattis sed. Suspendisse eu porta tellus. Cras gravida velit sed maximus fermentum. Fusce vitae metus commodo, sagittis nunc sed, faucibus nunc. Integer iaculis quam mattis, luctus neque in, viverra magna. Nulla rhoncus feugiat orci, quis posuere ligula ornare at. Integer vel sagittis risus. Donec semper metus nec finibus accumsan. Mauris sit amet suscipit ante. Aliquam accumsan, nisl vitae vulputate elementum, turpis nibh varius urna, vel bibendum nulla nunc ac quam. Aenean malesuada egestas maximus. Pellentesque faucibus, odio at tincidunt ullamcorper, eros nisi pellentesque mi, non blandit sapien neque quis lectus.
