# Supplementary material for: Prevalence of COVID-19 in adolescents and youth compared with older adults in states experiencing surges
Source: PLoS One. 2021 Mar 10;16(3):e0242587. doi: 10.1371/journal.pone.0242587 (PMC7946189; doi:10.1371/journal.pone.0242587)
Supplement: S1 Appendix — (ZIP) [file pone.0242587.s001.zip › S1_Appendix/page 2.pdf]

(iv) Florida: http://edr.state.fl.us/Content/populationdemographics/data/Pop_Census_Day.pdf

(v) Kansas: https://ipsr.ku.edu/sdc/region.php?area=Kansas&tab=1#TOTPOP

(vi) Missouri: https://healthapps.dhss.mo.gov/MoPhims/QueryBuilder?qbc=PNM&q=1&m=1

S1.3. New York Times Websites Indicating Surges by State:

(i) https://www.nytimes.com/interactive/2020/us/south-dakota-coronavirus-cases.html

(ii) https://www.nytimes.com/interactive/2020/us/tennessee-coronavirus-cases.html

(iii) https://www.nytimes.com/interactive/2020/us/utah-coronavirus-cases.html

(iv) https://www.nytimes.com/interactive/2020/us/florida-coronavirus-cases.html

(v) https://www.nytimes.com/interactive/2020/us/kansas-coronavirus-cases.html

(vi) https://www.nytimes.com/interactive/2020/us/missouri-coronavirus-cases.html

Since the data on the above websites are updated on a daily basis, we have included below in S1.4 the tables of the data the way they appeared on the websites when they were retrieved by us. These provide the numbers we used in our calculations.

S1.4. List of Supplemental Tables:

1) Table A, South Dakota COVID-19 Cases by Age (as of September 4, 2020);
2) Table B, Tennessee COVID-19 Cases by Age (as of August 12, 2020);
3) Table C, Utah COVID-19 Cases by Age (as of August 18, 2020);
4) Table D, Florida State COVID-19 Cases by Age (as of July 19, 2020);
5) Table E, Kansas COVID-19 Cases by Age (as 0f August 23, 2020);
6) Table F, Missouri COVID-19 Cases by Age (as of August 7, 2020);
7) Table G, South Dakota Demographics by Age;
8) Table H, Tennessee Population Counts by Age Group, Sex, Race, and Ethnicity, Estimates 2019;
9) Table I, Utah Demographics by Age, Sex, Race, Ethnicity;
10) Table J, Florida Population by Age Group;
11) Table K, Kansas Population by Age;
12) Table L, Missouri Population Demographics by Age.
