# Supplementary material for: Prevalence of COVID-19 in adolescents and youth compared with older adults in states experiencing surges
Source: PLoS One. 2021 Mar 10;16(3):e0242587. doi: 10.1371/journal.pone.0242587 (PMC7946189; doi:10.1371/journal.pone.0242587)
Supplement: S1 Appendix — (ZIP) [file pone.0242587.s001.zip › S1_Appendix/page 4.pdf]

Lorem Ipsum

Lorem ipsum dolor sit amet, consectetur adipiscing elit. Mauris maximus fringilla ligula, in malesuada erat tempor ac. Quisque dapibus posuere turpis, vel aliquam massa vehicula non.

TN Department of Health

Tennessee COVID-19 - August 12, 2020
Epidemiology and Surveillance Data

Total Cases:	126,393	Confirmed Cases:	124,391	Probable Cases:	2,002	Recovered Cases:	87,290
Total Deaths:	1,289	Confirmed Deaths:	1,249	Probable Deaths:	40		

Cases by County

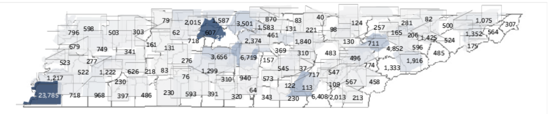

Daily Counts of Cases

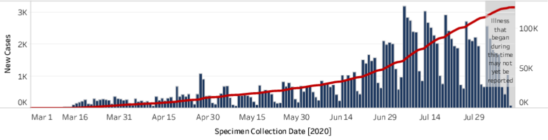

Demographics

Minimum Age	Average Age	Maximum Age
0	39	106

Age (years)	Cases	% of Cases	Deaths	% of Deaths
0-10	6,096	5%	4	0%
11-20	14,760	12%	1	0%
21-30	28,731	23%	14	1%
31-40	22,599	18%	24	2%
41-50	19,444	15%	72	6%
51-60	15,910	13%	137	11%
61-70	10,178	8%	259	20%
71-80	5,460	4%	376	29%
81+	3,007	2%	402	31%
Pending	208	0%	0	0%

Patient Sex	Cases	% of Cases	Deaths	% of Deaths
Female	63,587	50%	552	43%
Male	61,509	49%	736	57%
Pending	1,297	1%	1	0%

Race	Cases	% of Cases	Deaths	% of Deaths
American Indian or Alaska Native	160	0%	4	0%
White	58,694	46%	758	59%
Black or African American	24,484	19%	419	33%
Native Hawaiian or Other Pacific Islander	85	0%	2	0%
Asian	1,111	1%	16	1%
Other/Multiracial	17,778	14%	74	6%
Pending	24,081	19%	16	1%

Ethnicity	Cases	% of Cases	Deaths	% of Deaths
Not Hispanic or Latino	71,459	57%	1,165	90%
Hispanic or Latino	19,677	16%	106	8%
Pending	35,257	28%	18	1%

Outcomes

Ever Hospitalized	Cases	% of Cases
Yes	5,554	4%
No	69,079	55%
Pending	51,760	41%

Please note for hospitalizations: Information about hospitalization status is gathered at the time of diagnosis, therefore this information may be incomplete. This number indicates the number of patients that were *ever* hospitalized during their illness, it does not indicate the number of patients currently hospitalized.

Recovered	Cases	% of Cases
Yes	87,290	69%

Please note for recovered: TDH defines "recovered" as people who (1) have been confirmed to be asymptomatic by their local or regional health department and have completed their required isolation period or (2) are at least 21 days beyond the first test confirming their illness.

Deceased	Cases	% of Cases
Yes	1,289	1%
No	125,104	99%

Please note for deaths: Some deaths may be reported by health care providers, hospitals, medical examiners, local health departments, or others before they are included in the statewide count.

Laboratory Testing

Results	Number of Tests
PCR Positive	149,367
PCR Negative	1,608,323
Total	1,757,690

Please note, these data are meant to provide a rough estimate of testing volume. Due to different source data, numbers may differ slightly from day to day. Laboratory reports of positive cases are reported to metro and local health departments as soon as results are available. State numbers are updated at 2pm daily and there may be a lag in the reporting of cumulative numbers at the state level. Please note numbers within tables may not add up to total confirmed number of cases due to reporting delays and/or missing data. Percentages may not add up to 100.0% due to rounding.

Cases and Labs by County

County	Cases (Confirmed and Probable)	PCR Positive Tests	PCR Negative Tests	Active Cases	Recovered	Deaths
Anderson	711	845	15,799	237	468	6
Bedford	940	1,117	7,270	227	702	11
Benton	161	169	2,214	112	48	1
Bledsoe	717	739	4,793	66	649	2
Blount	1,333	1,566	16,460	456	862	15
Bradley	2,013	2,411	14,463	573	1,425	15
Campbell	257	293	6,286	113	143	1
Cannon	157	200	2,217	60	97	0
Carroll	341	359	4,340	227	109	5
Carter	564	623	9,257	417	141	6
Cheatham	607	728	6,735	277	321	9
Chester	230	253	1,951	100	128	2
Claiborne	281	312	5,749	115	166	0
Clay	83	85	1,475	34	49	0
Cocke	485	526	4,987	179	301	5
Coffee	573	674	9,846	302	268	3
Crockett	277	306	1,935	108	164	5
Cumberland	483	615	13,008	162	315	6
Davidson	21,044	26,107	196,867	3,774	17,049	221
Decatur	218	248	3,002	121	94	3
DeKalb	369	431	4,835	135	233	1
Dickson	718	895	8,126	360	355	3
Dyer	679	757	6,071	363	308	8
Fayette	718	903	10,590	257	453	8
Fentress	98	106	2,231	45	53	0
Franklin	343	424	7,099	124	215	4
Gibson	749	841	7,858	396	345	8
Giles	391	470	5,380	140	238	13
Grainger	206	242	3,200	83	123	0
Greene	524	610	8,547	354	161	9
Grundy	122	141	1,494	33	87	2
Hamblen	1,425	1,562	12,615	400	1,010	15
Hamilton	6,408	7,356	74,244	1,453	4,900	55
Hancock	82	92	1,206	55	25	2
Hardeman	968	1,089	8,946	363	587	18
Hardin	486	541	7,311	218	260	8
Hawkins	500	568	5,870	358	132	10
Haywood	522	554	3,624	287	228	7
Henderson	626	730	4,795	339	285	2
Henry	303	357	5,789	184	119	0
Hickman	276	351	3,453	116	160	0
Houston	62	73	1,267	28	34	0
Humphreys	131	151	3,203	78	50	3
Jackson	131	149	1,568	30	100	1
Jefferson	596	641	8,711	205	387	4
Johnson	307	319	4,010	262	45	0
Knox	4,852	5,446	79,532	1,825	2,987	40
Lake	796	971	4,524	79	717	0
Lauderdale	523	587	6,726	231	283	9
Lawrence	593	698	6,637	255	331	7
Lewis	76	84	2,389	46	29	1
Lincoln	320	393	4,653	142	177	1
Loudon	774	852	7,688	256	515	3
Macon	870	1,034	4,879	114	743	13
Madison	1,222	1,358	15,337	398	803	21
Marion	230	285	3,515	64	161	5
Marshall	310	356	4,903	128	178	4
Maury	1,299	1,521	13,260	543	748	8
McMinn	567	687	9,159	154	393	20
McNairy	397	449	3,840	188	203	6
Meigs	109	133	1,810	36	73	0
Monroe	458	520	5,433	188	261	9
Montgomery	2,015	2,331	25,677	1,042	959	14
Moore	64	78	987	29	35	0
Morgan	130	145	5,057	65	64	1
Obion	598	671	5,738	319	275	4
Overton	221	249	4,134	109	111	1
Perry	83	94	1,837	24	59	0
Pickett	40	41	672	20	19	1
Polk	213	232	1,983	66	143	4
Putnam	1,840	2,182	18,296	471	1,351	18
Rhea	547	655	5,937	102	443	2
Roane	496	587	10,943	226	268	2
Robertson	1,587	1,990	10,634	570	997	20
Rutherford	6,719	8,218	50,291	2,531	4,132	56
Scott	124	135	2,480	63	59	2
Sequatchie	113	137	3,042	32	80	1
Sevier	1,916	2,096	17,155	549	1,358	9
Shelby	23,785	29,135	248,667	4,060	19,410	315
Smith	461	519	3,449	150	307	4
Stewart	79	100	1,673	37	42	0
Sullivan	1,075	1,150	16,674	321	739	15
Sumner	3,501	4,227	26,188	1,277	2,151	73
Tipton	1,217	1,403	14,030	385	822	10
Trousdale	1,583	1,648	3,372	64	1,513	6
Unicoi	175	187	3,502	102	73	0
Union	165	183	2,615	79	86	0
Van Buren	37	40	789	12	25	0
Warren	545	603	8,016	251	290	4
Washington	1,352	1,537	17,686	954	396	2
Wayne	230	253	4,104	71	157	2
Weakley	503	538	6,014	354	145	4
White	310	363	4,863	141	164	5
Williamson	3,656	4,345	42,424	1,233	2,397	26
Wilson	2,374	2,845	24,252	799	1,552	23
Out of State	2,904	2,982	181,161	1,170	1,716	18
Pending	3,124	3,565	118,999	1,163	1,958	3

test-test-1 This is a preview of your figure rendered on a simulated PLOS journal page.

Maecenas ac est sit amet odio sollicitudin euismod. In risus odio, convallis a neque ac, varius ultricies arcu. Vestibulum et quam iaculis, ultricies odio et, molestie magna. Suspendisse vehicula purus id turpis eleifend, et convallis dui dignissim. Praesent tempus elit a metus sollicitudin, sed fringilla nulla porttitor. Nullam in tempus massa. Nunc maximus magna massa, nec volutpat risus rhoncus ut. Fusce quis ante sem. Aenean nulla nibh, tempus sit amet rhoncus at, eleifend vel risus. Sed dictum, sem ultrices elementum pharetra, lacus diam volutpat orci, scelerisque semper dui lacus ut enim.

Suspendisse in nunc id lacus commodo consequat. Proin semper aliquam varius. Fusce vitae neque aliquam nisi ultrices sodales vitae ut enim. Vivamus nec dictum ipsum. Sed condimentum ante eu urna tincidunt tincidunt. In ac lacus nec ipsum viverra volutpat posuere vel lacus. Class aptent taciti sociosqu ad litora torquent per conubia nostra, per inceptos himenaeos. Morbi rhoncus ipsum quis lorem hendrerit, at vulputate massa tempus. Ut arcu nisl, gravida vitae risus ultricies, porta venenatis massa. Cras dignissim, enim at faucibus aliquam, sapien nisl eleifend dolor, vel mollis nulla nisi id ipsum. Pellentesque vehicula ultricies risus sit amet faucibus. Praesent sit amet mi ac est faucibus accumsan. Praesent pulvinar sit amet orci auctor feugiat.
