# Supplementary material for: Prevalence of COVID-19 in adolescents and youth compared with older adults in states experiencing surges
Source: PLoS One. 2021 Mar 10;16(3):e0242587. doi: 10.1371/journal.pone.0242587 (PMC7946189; doi:10.1371/journal.pone.0242587)
Supplement: S1 Appendix — (ZIP) [file pone.0242587.s001.zip › S1_Appendix/page 8.pdf]

Lorem Ipsum

Lorem ipsum dolor sit amet, consectetur adipiscing elit. Mauris maximus fringilla ligula, in malesuada erat tempor ac. Quisque dapibus posuere turpis, vel aliquam massa vehicula non.

Table F. Missouri COVID-19 Cases by Age (as of August 07, 2020)

Age	Number of Cases
0-9	1677
10-19	6569
20	1607
21	1644
22	1558
23	1550
24	1504
25	1422
26	1431
27	1404
28	1360
29	1327
30	1265
31	1180
32	1189
33	1095
34	1116
35	1094
36	1010
37	1055
38	1066
39	1038
40	1052
41	922
42	984
43	958
44	936
45	980
46	924
47	972
48	995
49	1044
50	1004
51	1016

test-test-1 This is a preview of your figure rendered on a simulated PLOS journal page.

Maecenas ac est sit amet odio sollicitudin euismod. In risus odio, convallis a neque ac, varius ultricies arcu. Vestibulum et quam iaculis, ultricies odio et, molestie magna. Suspendisse vehicula purus id turpis eleifend, et convallis dui dignissim. Praesent tempus elit a metus sollicitudin, sed fringilla nulla porttitor. Nullam in tempus massa. Nunc maximus magna massa, nec volutpat risus rhoncus ut. Fusce quis ante sem. Aenean nulla nibh, tempus sit amet rhoncus at, eleifend vel risus. Sed dictum, sem ultrices elementum pharetra, lacus diam volutpat orci, scelerisque semper dui lacus ut enim.

Suspendisse in nunc id lacus commodo consequat. Proin semper aliquam varius. Fusce vitae neque aliquam nisi ultrices sodales vitae ut enim. Vivamus nec dictum ipsum. Sed condimentum ante eu urna tincidunt tincidunt. In ac lacus nec ipsum viverra volutpat posuere vel lacus. Class aptent taciti sociosqu ad litora torquent per conubia nostra, per inceptos himenaeos. Morbi rhoncus ipsum quis lorem hendrerit, at vulputate massa tempus. Ut arcu nisl, gravida vitae risus ultricies, porta venenatis massa. Cras dignissim, enim at faucibus aliquam, sapien nisl eleifend dolor, vel mollis nulla nisi id ipsum. Pellentesque vehicula ultricies risus sit amet faucibus. Praesent sit amet mi ac est faucibus accumsan. Praesent pulvinar sit amet orci auctor feugiat.
