# Supplementary material for: Prevalence of COVID-19 in adolescents and youth compared with older adults in states experiencing surges
Source: PLoS One. 2021 Mar 10;16(3):e0242587. doi: 10.1371/journal.pone.0242587 (PMC7946189; doi:10.1371/journal.pone.0242587)
Supplement: S1 Appendix — (ZIP) [file pone.0242587.s001.zip › S1_Appendix/page 9.pdf]

Lorem Ipsum

Lorem ipsum dolor sit amet, consectetur adipiscing elit. Mauris maximus fringilla ligula, in malesuada erat tempor ac. Quisque dapibus posuere turpis, vel aliquam massa vehicula non.

Age	Number of Cases
52	966
53	1005
54	935
55	989
56	945
57	1008
58	989
59	912
60	911
61	863
62	822
63	827
64	793
65	674
66	673
67	576
68	592
69	577
70	554
71	513
72	524
73	467
74	401
75	392
76	374
77	421
78	360
79	335
80-84	1421
85-89	1224
90+	1254
Unknown	172

From: https://health.mo.gov/living/healthcondiseases/communicable/novel-coronavirus/cases-by-age.php

Appendix S1_202102222145-9.tif This is a preview of your figure rendered on a simulated PLOS journal page.

Maecenas ac est sit amet odio sollicitudin euismod. In risus odio, convallis a neque ac, varius ultricies arcu. Vestibulum et quam iaculis, ultricies odio et, molestie magna. Suspendisse vehicula purus id turpis eleifend, et convallis dui dignissim. Praesent tempus elit a metus sollicitudin, sed fringilla nulla porttitor. Nullam in tempus massa. Nunc maximus magna massa, nec volutpat risus rhoncus ut. Fusce quis ante sem. Aenean nulla nibh, tempus sit amet rhoncus at, eleifend vel risus. Sed dictum, sem ultrices elementum pharetra, lacus diam volutpat orci, scelerisque semper dui lacus ut enim.

Suspendisse in nunc id lacus commodo consequat. Proin semper aliquam varius. Fusce vitae neque aliquam nisi ultrices sodales vitae ut enim. Vivamus nec dictum ipsum. Sed condimentum ante eu urna tincidunt tincidunt. In ac lacus nec ipsum viverra volutpat posuere vel lacus. Class aptent taciti sociosqu ad litora torquent per conubia nostra, per inceptos himenaeos. Morbi rhoncus ipsum quis lorem hendrerit, at vulputate massa tempus. Ut arcu nisl, gravida vitae risus ultricies, porta venenatis massa. Cras dignissim, enim at faucibus aliquam, sapien nisl eleifend dolor, vel mollis nulla nisi id ipsum. Pellentesque vehicula ultricies risus sit amet faucibus. Praesent sit amet mi ac est faucibus accumsan. Praesent pulvinar sit amet orci auctor feugiat.

Phasellus vitae congue est. Duis rutrum iaculis nunc, sed sollicitudin neque eleifend nec. Pellentesque ac nisi eget tortor imperdiet sagittis ut in orci. Mauris porta convallis euismod. Donec in ultricies urna, nec interdum lectus. Nullam sit amet finibus augue, eget rutrum metus. Nam faucibus, urna ac finibus eleifend, neque nisi lobortis ante, at pharetra purus purus sed urna. Curabitur sit amet dui at enim porta posuere non vehicula ligula. Suspendisse potenti. Vestibulum arcu magna, vulputate a massa ac, molestie tincidunt dui.

Donec id tempus lacus, sed tristique nulla. Nullam rutrum risus ut pharetra porttitor. Nam mattis dolor erat, sed volutpat est mattis sed. Suspendisse eu porta tellus. Cras gravida velit sed maximus fermentum. Fusce vitae metus commodo, sagittis nunc sed, faucibus nunc. Integer iaculis quam mattis, luctus neque in, viverra magna. Nulla rhoncus feugiat orci, quis posuere ligula ornare at. Integer vel sagittis risus. Donec semper metus nec finibus accumsan. Mauris sit amet suscipit ante. Aliquam accumsan, nisl vitae vulputate elementum, turpis nibh varius urna, vel bibendum nulla nunc ac quam. Aenean malesuada egestas maximus. Pellentesque faucibus, odio at tincidunt ullamcorper, eros nisi pellentesque mi, non blandit sapien neque quis lectus.
